# Prevalence of high-risk human papilloma virus in women with high-grade squamous cell intraepithelial lesions in Botswana using Abbott Real*Time* HPV assay

**DOI:** 10.1371/journal.pone.0211260

**Published:** 2019-01-30

**Authors:** Patricia Rantshabeng, Ishmael Kasvosve, Andrew Ndlovu, Simani Gaseitsiwe, Sikhulile Moyo

**Affiliations:** 1 Department of Medical Laboratory SciencesFaculty of Health Sciences, University of Botswana, Gaborone, Botswana; 2 Botswana-Harvard AIDS Institute Partnership, Gaborone, Botswana; 3 Department of Immunology & Infectious Diseases, Harvard T.H. Chan School of Public Health, Boston, MA, United States of America; Istituto Nazionale Tumori IRCCS Fondazione Pascale, ITALY

## Abstract

**Background:**

High-risk human papillomavirus (HR-HPV) has been demonstrated to be the necessary cause of cervical carcinoma. High-risk HPV detection has a prognostic significance for the women who are at increased risk of disease progression. HPV genotyping in cervical cancer precursor lesions is crucial for prevention and management of cervical cancer. This study was designed to investigate the distribution of HR-HPV genotypes among a group of patients with high-grade squamous intraepithelial lesions and higher, of the cervix, in Botswana.

**Materials and methods:**

185-archived residual formalin-fixed paraffin-embedded cervical biopsies collected between the years, 2006 and 2008 were studied. These tissues were diagnosed with HSIL (n = 146) and squamous cell carcinoma (n = 39). DNA was extracted using the Abbott m2000 analyser (Abbott Laboratories, Illinois) using reagents provided by the manufacturer. HPV genotyping was done using the Abbott Real*Time* HR-HPV PCR, which qualitatively detects 14 HR-HPV (reported as HPV 16, 18 & Other HR-HPV).

**Results:**

DNA was successfully extracted from 162/185 (87.6%) tissues as indicated by a positive β-globin test. 132/162 (82%) tested positive for HR-HPV The HPV 16 prevalence was 50% (66/132), HPV 18 at 15.2% (20/132) and other Group 1 HR-HPV plus HPV 66 and 68 had a prevalence of 56.1% (74/132). Other HR-HPV types were common in HSIL than in carcinoma, while HPV 16 was more prevalent in carcinomas than other HR-HPV genotypes.

**Conclusion:**

In this study, HPV 16 and other HR-HPV genotypes were commonly associated with HSIL but HPV 18 was uncommon among Botswana women. Our data highlights the need for multivalent HPV vaccines with cross coverage for other high risk HPV other than HPV 16 and 18.

## Introduction

Human papillomaviruses (HPV) are sexually transmitted and mucotropic and the oncogenic subset termed “high-risk” (HR-HPV) types, has been reported to be the necessary causal agents for the development of cervical carcinoma and its precursor lesions [1–3] Cervical cancer development has been reported to be virtually impossible in the absence of HR-HPV infection [4]. To date, over 120 HPV types have been identified and are classified as either high-risk (HR) or low-risk (LR) based on their association with cervical cancer [1, 3].

Identified HR-HPV types include HPV 16, 18, 31, 33, 35, 39, 45, 51, 52, 58, 59, 68, 73 and 82 [1]; although HPV type 68 has been found to have limited evidence in human cancers but strong mechanistic implications & HPV types 73 and 82 have also been evaluated as having limited evidence in cervical cancer (Bouvard et al., 2009). The 14 HPV genotypes are categorized as high-risk (HR) because of their association with virtually all cases of cervical cancer but HPV types 16 and 18 are the most commonly isolated in studies worldwide (Strickler et al., 2005; Alam et al., 2008). The low-risk HPV types include HPV 6, 11, 40, 42, 43, 44, 54, 61, 70, 72, 81 and 89 and mainly cause genital warts and oral papillomas [1, 4]

Among asymptomatic women, the global cervical HPV prevalence is estimated at 10.4% and it varies according to geographical region, ranging from a low of 6.2% in south-eastern Asia to a high of 31.6% in east Africa [5]

The high-risk HPV prevalence increases with increasing severity of disease and in studies in Africa the prevalence ranged from 22% in normal cytology to 90% in invasive cervical cancer [6] and approximately 63±2% of invasive cervical cancers in this region were attributed to HPV type 16 or 18 **(**Denny, 2014**)**. Cervical cancer is a leading cause of cancer-related deaths in women in Botswana and about two-thirds of the deaths occur in HIV-infected women [7]. Botswana has a high rate of cervical cancer with an age-standardized incidence of 38/100,000 women and this is largely driven by the HIV burden in the population [8]. The prevalence of HIV in Botswana in the 15–49 year age group is 17% but is estimated to be 24% in pregnant women [9].

It is estimated that there are approximately 602 000 women aged 15 years and older in Botswana, are at risk of developing cervical cancer and about two-thirds of those at risk are aged 15–44 years [10]. World Health Organization (WHO) has predicted that by year 2025, mortality from cervical cancer in Botswana will increase by more than 20% in women under the age of 65 years, if the current trend continues (Health-Botswana, 2012). While HPV is known to be the most common sexually transmitted viral disease in the developed countries, it is currently the least known sexually transmitted infection among the general population.

High-grade squamous cell intraepithelial lesion (HSIL) is a stage in cervical carcinogenesis that immediately precedes cancer and it is characterized by HPV persistence [11]. It is considered a pre-cancer and the best predictor for cancer risk. It is also a critical endpoint for evaluating the efficacy of primary and secondary prevention and treatment strategies [12]. The aim of this study was to determine the prevalence of HR-HPV in formalin-fixed paraffin embedded (FFPE) cervical tissue biopsies diagnosed with HSIL.

## Materials and methods

### Study population

A total of 185-archived residual formalin-fixed paraffin-embedded (FFPE) cervical biopsies collected between the years 2006 and 2008, and processed at Botswana’s National Health Laboratory (NHL), were included in this analysis. The tissues that were diagnosed with HSIL and cervical squamous cell carcinoma by a pathologist. Participants’ age, HIV-infection status, Pap test results (from 2006–2008), and histopathology findings were abstracted from the medical record. The study was approved by the University of Botswana’s Institutional Review Board and Health Research and Development Unit (HRDC) of the Ministry of Health & Wellness. Samples were uniquely identified and de-linked to protect the patient information during the study.

### Tissue sectioning and de-waxing

Formalin-fixed and paraffin- embedded (FFPE) tissues were aseptically sectioned at 20μm into 15ml centrifuge tubes for de-waxing and re-hydration before HR-HPV DNA extraction and genotyping. Tissue sections were incubated in 15 ml of xylene at room temperature for one hour. This step was repeated three times to ensure maximal removal of paraffin wax. The tissue sections were then incubated in absolute alcohol and left to stand at room temperature for one hour. The step was repeated twice for effective removal of xylene. The tissue sections were taken through various steps of de-graded alcohol to introduce water to tissue, starting with 95%, then 80%, and 70% alcohols. Finally the tissues were washed with Phosphate Buffered Saline and kept in distilled water overnight.

### Tissue digestion

Tissues were transferred to well labelled heat resistant cryovials, 200μl of Qiagen Proteinase K (Thermo Fisher Scientific, Waltham, MA, USA) and 600μl lysis buffer (bioMerieux SA Marcy E’Toile, France) were added to the tissue samples for overnight digestion in a 56°C water bath. This process was terminated by further incubation of tissue digest at 100°C for one hour.

### DNA extraction and high-risk HPV genotyping

After tissue digestion, samples were centrifuged at 3500 rpm for 5 minutes and 700μl of the supernatant was transferred into barcoded Abbott m2000 PCR tubes, loaded into the Abbott m2000 machine for DNA extraction, amplification and HR-HPV genotyping using the Abbott Real*Time* HR-HPV assay (Huang et al, 2009; Tang N, et al, 2009). This is an automated process that uses micro beads technology for DNA extraction. The assay is a qualitative in-vitro test that amplifies and detects HR-HPV DNA in cervical cells. Detection of all 14 HR-HPV genotypes (HPV 16, 18, 31, 33, 35, 39, 45, 51, 52, 56, 58, 59, 66, and 68) was achieved through a primer mix targeting the conserved L1 region of HR-HPV genomes and single stranded DNA probes. This assay also uses human-β-globin as an internal control (IC), the negative control (NC) and the positive control (PC). The IC is used to confirm the sample adequacy, DNA extraction, success of amplification and to indicate whether there were any PCR inhibitors present in the sample.

The controls were run in parallel with the samples and the IC results were reported parallel to HR-HPV results in a sample. Any sample that failed the IC test automatically failed the DNA extraction process and had to be repeated. This assay can only differentiate between HPV 16, HPV 18 types; non-HPV 16/18 genotypes are reported as ‘Other HR-HPV’. Results were reported and analysed as a dichotomous variable: “HR-HPV detected” or “NO HR-HPV DETECTED” along with the genotype detected for those that are positive for HR-HPV [13]

### Statistical analysis

Prevalence estimates are presented with 95% confidence intervals calculated using the binomial exact methods. A p-value of 0.05 and less was considered to be statistically significant. We compared prevalence of HR-HPV genotypes according to cervical lesions using a Fisher’s exact test and p-value <0.05 was considered significant. Analysis was performed using STATA version 14.2 (College Station, Texas) using a Fisher’s exact test and p-value <0.05 was considered significant. Analysis was performed using STATA version 14.2 (College Station, Texas).

## Results

Out of 185 FFPE tissues enrolled, amplifiable DNA extraction was successfully achieved on 162 tissue blocks (87.6%) while 23 tissues failed and were excluded from further testing. Amplifiable DNA was defined as that with a positive internal control (human β-globin). Out of the 162 tissue blocks, 124 showed HSIL and 38 had carcinoma. Women had a median age of 38 years (Q1,Q3: 33, 44), and at least 44% were HIV infected (71/162) (**Table 1).**

**Table 1:** Characteristics of the study participants with successful DNA amplification.

Characteristic	
Age, years (Median (Q1, Q3) (n = 157)	38 (33, 44)
Cervical lesion type (n = 162)	n (%)
High-grade squamous cell intraepithelial lesion	124 (76.5%)
Squamous Cell Carcinoma	38 (23.5%)
HIV status (n = 162)	n (%)
HIV negative	4 (2.5%)
HIV positive	67 (41.1%)
Undocumented	91 (56.4%)

High-risk HPV prevalence was 81.5% (132/162) (**Table 2)**. Single-type infections were more common than multi-type, especially for HPV 16. Among those with hr-HPV, the proportion with HPV 16, 18 and other HR-HPV types were 36.4%, 4.5% and 46.9%, respectively. Among those multiple hr-HPV tests positive, HPV 16+other HR-HPV combination (6%) was the most common (Table 2).

**Table 2:** Distribution of high-risk human papillomavirus (HPV) genotypes in the study.

HPV type	Number of women affected	Proportion with HPV (95% CI)
All hr-HPV (n = 162)	132	81.5% (74.6–87.1)
Single Test Positive among hr-HPV (n = 132)		
HPV 16	48	36.4% (28.2–45.2)
HPV 18	6	4.5% (1.7–9.6)
Other hr-HPV	56	42.2% (33.9–51.3)
Multiple Tests Positive among hr-HPV (n = 132)		
HPV 16+18	4	3.0% (0.8–7.6)
HPV 16+other hr-HPV	8	6.0% (2.7–11.6)
HPV 18+other hr-HPV	4	3.0% (0.8–7.6)
HPV 16+18+other hr-HPV	6	4.5% (1.7–9.6)

CI–confidence interval; HPV—human papilloma virus; hr-HPV–high-risk human papilloma virus

Other HR-HPV genotypes were most prevalent in HSIL at 50.1% (56/110) as compared to HPV 16 at 36.4% (40/110) and HPV 18, at 3.6% (4/110). Human papillomavirus type 16 was more prevalent at 36.4% (8/22), compared to other HR-HPV at 27.3%, (6/22) and HPV 18, 2/22 (9.1%) in carcinomas (**Table 3).** However, there was no statistically significant differences observed in proportions with HPV 16, HPV 18 and other HR-HPV prevalence across the two cervical lesions studied except for multiple infections (p = 0.03) (**Table 3**).

**Table 3:** Comparison of high-risk human papillomavirus genotype distributions according to cervical lesions.

HPV type	Cervical Lesion Type				P-value
	HSIL% (95% CI) n = 110		Carcinoma% (95% CI) n = 22		
HPV 16	36.4%	27.4–46.1	36.4%	(17.2–59.3)	1.0
HPV 18	3.6%	0.1–7.1	9.1%	(7.1–9.9)	0.26
Other hr-HPV	50.2%	40.3–59.7	27.3%	(10.7–50.2)	0.06
Multiple hr-HPV infection	9.1%	8.4–9.6	27.3%	(10.7–50.2)	0.03

CI–confidence interval; HPV—human papilloma virus; hr-HPV–high-risk human papilloma virus

## Discussion

The prevalence of HR-HPV genotypes for women in Botswana has not been reported before and studies on the HR-HPV genotype distribution in HIV-uninfected women in Botswana are lacking. Previous studies have reported HR-HPV prevalence in Botswana women with HIV infection diagnosed with abnormal cytology and histology [14, 15]. It is estimated that up to 90% of women with cervical intraepithelial lesions are HPV-DNA positive [16, 17]. The presence of cervical HPV DNA is often associated with cytological and histological changes of the cervical epithelium [11].

Overall, the HR-HPV prevalence was 81.5% in HSIL and cancer tissues obtained from one regional referral hospital in Botswana. The prevalence rate in the current study is lower than previously reported in another study in Botswana in which 92% of the study participants had HR-HPV infection, but were also co-infected with HIV [15]. Also, the observed lower prevalence might be due to the limitation of our approach. Using the sandwich procedure and testing the centre section might improve detection as previously reported[18]. HSIL, although uncommon in < 30 years, has been reported in women in their 20’s with onset of sexual relationships.

In our study of women with HSIL and cervical cancer, there were fewer women younger than 30 years. Botswana, like in many developing countries, the majority of cervical cancer cases are diagnosed at a late stage [19] and this may explain why there were fewer younger women with cervical lesions (<30 years) in our study. Furthermore, in Botswana cervical cancer screening is offered to women aged ≥30 years.

In this study, data on HIV status of the participants was incomplete. Studies conducted in sub-Saharan Africa have shown a high prevalence of HR-HPV in patients with HIV co-infection [20]. Our results are also comparable to those of other studies done elsewhere, in the African region. A study done in Tanzania on a similar population of women, reported an HR-HPV prevalence rate of 88% [21]. Similar studies conducted in Mali and Senegal reported HR-HPV prevalence rates of 88.1% and 86.5% respectively, in patients with HSIL [22]. In Europe, a study done on African women with HIV infection reported a 43% prevalence of HR-HPV [23].

A study done in Thailand on women with HSIL, reported a high prevalence of 96.97% [24]. In the US, a study done by Hariri *et al*., [25, 26] also reported 95% HR-HPV prevalence in women with HSIL in North Carolina. A similar study [25] also reported high HR-HPV (95.4%) prevalence in women with pre-cervical lesions.

HPV types 16 and 18 infections are associated with greater risk for cervical disease progression [27]. Single HPV 16 infection accounted for 36.4% (95% CI 28.2–45.2) and 42.2% (95% CI 33.9–51.3) of the all cases were due to other HR-HPV types. Less than 5% of the cases were due to HPV 18 single-infection. Multiple HR-HPV co-infections accounted for less than 20% of all the cases, **Table 2**. HPV 18 prevalence was lowest in our study as compared to HPV 16 and other HR-HPV types. A lower prevalence of HPV 18 has been observed in other similar studies from around the world. In North Carolina, United States, the HPV 18 prevalence in a study of women with pre-cancer lesions was 9% [25]. The prevalence of HPV 16 and other HR-HPV types were high in the population we studied. A similar study in North Carolina, reported a higher prevalence of HPV 16 followed by HPV 52 and 18 [26].

Another study in the US, reported a lower HPV 18 prevalence that is similar to our findings [25]. In Nigeria, HPV 16 and other types, (HPV 35, 58 and 31) were the most prevalent types [28]. In Tanzania, the most common HPV types reported in HSIL were HPV 53 and 58 [21]. The same findings were reported by a study in Thailand of women with HSIL where HPV 16 and 58 were reported as the most prevalent compared to HPV 18 [24]. In Botswana, another study reported HPV 58 to be the most prevalent in HSIL, [14]. In our study, we were unable to delineate the other 12 HR-HPV genotypes.

These data contribute evidence that although HPV 16 was commonly isolated in this study, HPV 18 was rare as compared to findings in other regions of the world. Other HR-HPV types were the most prevalent and had a higher distribution among women with HSIL. This has implications on HPV vaccination for prevention of cervical cancer. To date, there is no known cure for HPV infection and prevention via vaccination remains the bulwark against some selected HR-HPV types.

In 2011, MacLeod et al.,[14] highlighted the importance of investigating HPV types besides HPV 16 and 18, that could have a possible impact on the current vaccination designs for the HIV infected women population in Botswana [14]. Their study found HPV type 58 to be the most prevalent compared to HPV types 16 and 18. Another study also reported a high prevalence of other HR-HPV genotypes (HPV 33, 35 and 58) in HIV-infected women with CIN 2/3 [29] and based on these studies, the current vaccine design used in Botswana might only prevent half of the HR-HPV-associated cervical neoplasias.

When we compared HR-HPV genotypes according to clinical lesions, there was no difference in the distribution of HPV 16, 18 or other HR-HPV (p ≥ 0.06). However, we observed a difference in in the distribution of multiple HR-HPV types according to cervical lesions, p = 0.03 (Table 3), but these observation is based on a small sample size.

Molecular testing of HPV genotypes also has the potential to provide an alternative, more sensitive and efficient strategy for cervical cancer risk screening and prevention than do methods based solely on cytology. In our study, few cytology results reported HPV infection and the majority did not compared to histology reports that reported HPV effects. Despite exclusion of HPV reporting, most HSIL tissues tested positive for HR-HPV with PCR. Determining the prevalence of HR-HPV for any location is important in monitoring HR-HPV -associated cervical lesions and informing cervical cancer prevention strategies [30].

Our study has several limitations. Firstly, the Abbott Real*Time* HR-HPV detection assay used cannot differentiate the non-HPV 16 and 18 HR-HPV genotypes. Therefore, we are unable to conclude if all the Group I HR-HPVs were present, accounting for the 56.1% prevalence obtained in the study. While the Abbott Real*Time* HR-HPV is a widely used real-time PCR assay with high sensitivity and specificity, some studies have observed that the assay has been less sensitive for a few cases of HPV16 when HPV16 is present in co-infection with other HR-HPVs types, probably related to low HPV16 viral load below the Abbott Real*Time* HR HPV assay cut-off [31, 32].

There is also limited data on literature regarding the concordance between the Abbott HR-HPV assays performed on paraffin-embedded tissue specimens as compared to data on fresh cytology specimens. However, the performance of the Abbott Real*Time* HPV test is comparable to conventional nested PCR in cervical smear specimens for detection of HPVs associated with high-grade lesions [33]. We used archived tissue samples and some clinical data e.g. HIV status was not available on all patients. Consequently, we were unable to explore the interaction of HR-HPV with HIV as reported in other studies. Nevertheless, data from this study is an important contribution to the understanding of cervical cancer precursor lesions and HR-HPV prevalence in Botswana.

## Conclusions

Human papillomavirus type 16 and other HR-HPV genotypes were commonly associated with HSIL but HPV 18 was uncommon among Botswana women evaluated at this regional hospital. Vaccines offering protection against HPV 16 and 18 only, may provide partial protection for women in our study population in Botswana with a high prevalence of non-HR-HPV 16 and 18. However, whether newer vaccine strategies (HPV4 and HPV9 vaccines) can provide additional protection against other HR-HPV was not evaluated in this study.
